# Increased Use of Bayesian Network Models Has Improved Environmental Risk Assessments

**DOI:** 10.1002/ieam.4369

**Published:** 2020-12-11

**Authors:** S Jannicke Moe, John F Carriger, Miriam Glendell

**Affiliations:** † Norwegian Institute for Water Research (NIVA), Oslo, Norway; ‡ United States Environmental Protection Agency, Office of Research and Development, Center for Environmental Solutions and Emergency Response, Land Remediation and Technology Division, Environmental Decision Analytics Branch, Cincinnati, Ohio; § James Hutton Institute, Craigiebuckler, Aberdeen, United Kingdom

**Keywords:** Bayesian networks, Ecological risk assessment, Environmental risk assessment, Probabilistic modeling, Causal modeling

## Abstract

Environmental and ecological risk assessments are defined as the process for evaluating the likelihood that the environment may be impacted as a result of exposure to stressors. Although this definition implies the calculation of probabilities, risk assessments traditionally rely on nonprobabilistic methods such as calculation of a risk quotient. Bayesian network (BN) models are a tool for probabilistic and causal modeling, increasingly used in many fields of environmental science. Bayesian networks are defined as directed acyclic graphs where the causal relationships and the associated uncertainty are quantified in conditional probability tables. Bayesian networks inherently incorporate uncertainty and can integrate a variety of information types, including expert elicitation. During the last 2 decades, there has been a steady increase in reports on BN applications in environmental risk assessment and management. At recent annual meetings of the Society of Environmental Toxicology and Chemistry (SETAC) North America and SETAC Europe, a number of applications of BN models were presented along with new theoretical developments. Likewise, recent meetings of the European Geosciences Union (EGU) have dedicated sessions to Bayesian modeling in relation to water quality. This special series contains 10 articles based on presentations in these sessions, reflecting a range of BN applications to systems, ranging from cells and populations to watersheds and national scale. The articles report on recent progress in many topics, including climate and management scenarios, ecological and socioeconomic endpoints, machine learning, diagnostic inference, and model evaluation. They demonstrate that BNs can be adapted to established conceptual frameworks used to support environmental risk assessments, such as adverse outcome pathways and the relative risk model. The contributions from EGU demonstrate recent advancements in areas such as spatial (geographic information system [GIS]–based) and temporal (dynamic) BN modeling. In conclusion, this special series supports the prediction that increased use of Bayesian network models will improve environmental risk assessments.

## EDITOR’S NOTE

This article is part of the special series “Applications of Bayesian Networks for Environmental Risk Assessment and Management” and was generated from a session on the use of Bayesian networks (BNs) in environmental modeling and assessment in 1 of 3 recent conferences: SETAC North America 2018 (Sacramento, CA, USA), SETAC Europe 2019 (Helsinki, Finland), and European Geosciences Union 2019 (Vienna, Austria). The 3 sessions aimed at showing the state-of-the art and new directions in the use of BN models in environmental assessment, focusing on ecotoxicology and water quality modeling. This series aims at reflecting the broad applicability of BN methodology in environmental assessment across a range of ecosystem types and scales, and discusses the relevance for environmental management.

## INTRODUCTION

**Figure 1.**
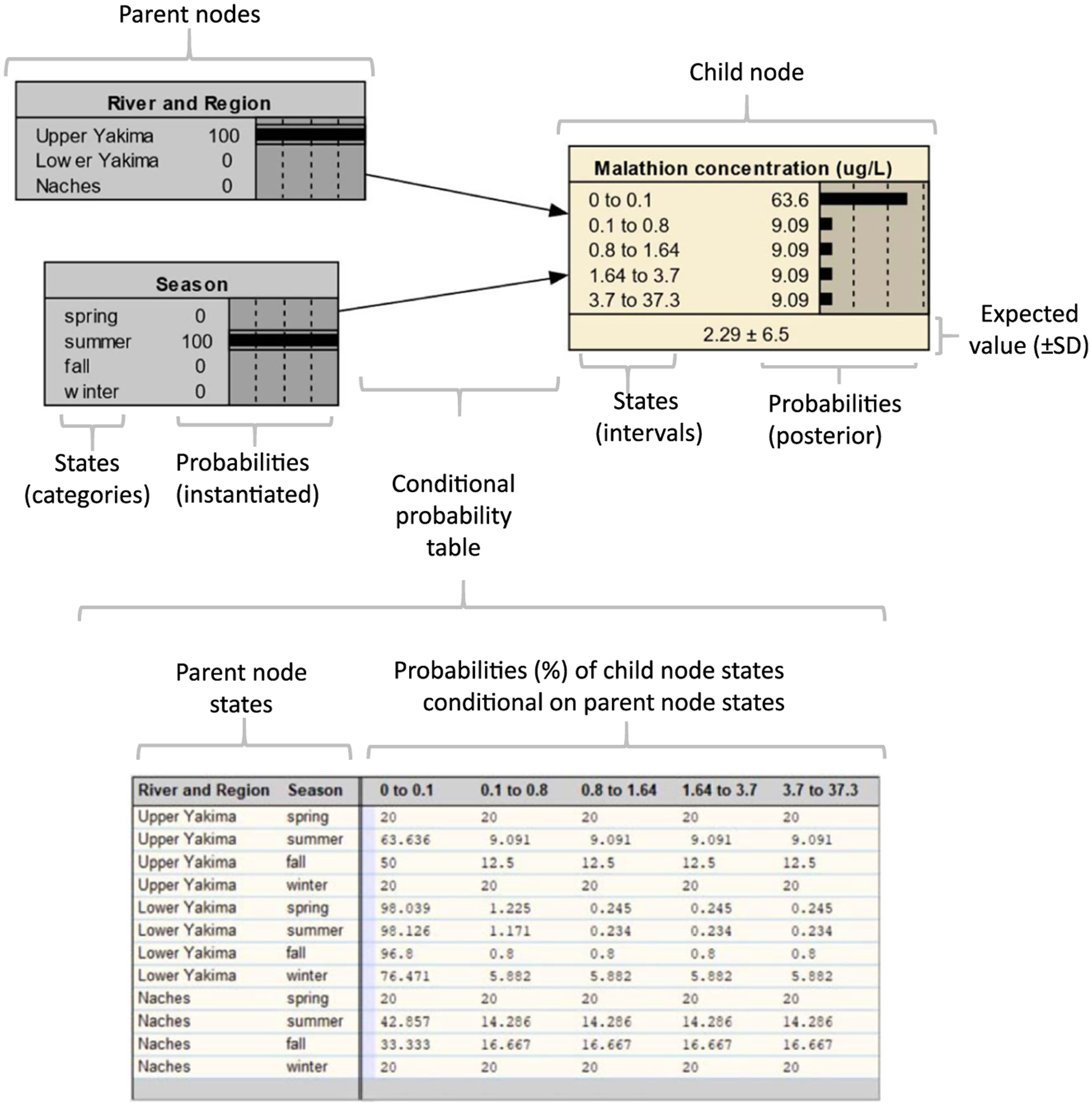
Main components of a Bayesian network (from Mitchell et al. this issue). The full model is described in Figure 2. The nodes represent probability distributions over mutually exclusive states. The relationship between parent and child nodes is quantified by a conditional probability table, representing the probability of each child node state given each combination of parent node states.

“Increased use of Bayesian network models will improve ecological risk assessments” was the title of an editorial paper by Hart and Pollino (2008), which documented an increase in Bayesian network (BN) model applications with relevance for ecological risk assessment. Readers not yet familiar with BN models may not find this statement informative, but hopefully intriguing enough to explore this special series on applications of BNs in environmental risk assessment and management. In brief, BNs are graphical, probabilistic, and causal models (Figure 1). They can combine qualitative knowledge and quantitative data to model a system using probabilities, which is consistent with the concept of risk being probabilistic (Pollino et al. 2007). The process of risk assessment is formally defined in terms of likelihood or probability. For example, the United States Environmental Protection Agency defines ecological risk assessment as “the process for evaluating how likely it is that the environment may be impacted as a result of exposure to one or more environmental stressors…” (USEPA 2020). The European Food Safety Authority defines risk characterization as “the final stage of risk assessment, in which the likelihood that a particular substance will cause harm is calculated in the light of the nature of the hazard and the extent to which people, animals, plants and/or the environment are exposed to it” (EFSA 2020). (The term “ecological risk assessment” is commonly used in North America whereas the term “environmental risk assessment” in more commonly used in Europe. In the present paper, we use the term “environmental risk assessment” [ERA] to include both ecological and human health endpoints [EEA 1998]). Although probabilistic methods have been recommended for risk assessment (Jager et al. 2001; USEPA 2014; Van den Brink et al. 2016), they are not yet thoroughly implemented in regulatory risk frameworks. In practice, it is still more common to use so-called “deterministic” approaches, such as the single-value risk quotient calculated as the ratio of predicted exposure and no-effect concentrations of a stressor (Fairbrother et al. 2016).

This special series of 10 articles can be viewed as a follow-up and evaluation of the statement by Hart and Pollino (2008). The series starts with a literature review (Kaikkonen et al. this issue), which documents the increased use of BNs in the context of ERA over the last 15 y. This is followed by 7 articles with case studies from different environmental domains, which exemplifies recent advancements in BN modeling and improvements for ERA. Three of these case studies apply the BN relative risk model; the history and development of this modeling framework for ERA is documented in a separate paper (Landis this issue). Finally, the series provides discussion and recommendations on how to handle uncertainty in BNs for risk assessment (Sahlin et al. this issue). This series of papers demonstrates that increased use of BN models has indeed improved and will continue to improve ERAs.

## BAYESIAN NETWORKS SESSION IN SETAC ANNUAL MEETINGS

To facilitate the discussion of the usage of BNs for risk assessment, conference sessions focusing on BN modeling were initiated by the authors in the Society of Environmental Toxicology and Chemistry (SETAC) annual meetings since 2018, first in North America (chaired by WG Landis and JF Carriger) and subsequently in Europe (by SJ Moe, WG Landis, and DN Barton). The sessions attracted international scientists and provided diverse perspectives on the application of BNs. Presenters and attendees were from multiple career stages and experience levels, and some of the most enthusiastic participants in these sessions were those who were new to BNs. Discussions in these sessions were often focused on lessons learned and identifying where BNs will benefit the development and application of risk assessment. The authors of the manuscripts in this special series provide a cross-section of the presenters that were involved with these sessions. Concurrently, the European Geosciences Union (EGU) organized sessions addressing the application of Bayesian approaches more generally in water quality modeling (chaired by M Glendell). We invited selected papers from the EGU session in 2019 because practitioners of BN modeling in ecotoxicology can learn from the recent developments in this closely related field.

The *Integrated Environmental Assessment and Management* editorial team has already recognized this modeling approach by publishing the previous special series “Bayesian Networks in Environmental and Resource Management” (Barton et al. 2012), which addressed environmental management applications more broadly. The present series focuses more specifically on the applicability of BNs as a probabilistic and integrative modeling method for ERA and as an alternative to the more traditional deterministic methods. As shown in the articles of this special series, the application of BNs to ERAs continues to grow along with the complexity of problems that must be tackled by ERA, from a changing climate to multiple stressors and scales (Landis et al. 2013). The uncertainties and information requirements for ERA are only increasing, and the present special series comes at a critical time for the application and development of BNs in ERAs.

Although BNs are becoming more of a known quantity, their mainstream acceptance by the ERA community is still largely scarce. In our experience, this is not due to an inherent problem with the BN methodology or its usefulness, but an unfamiliarity and mystification of what it is. Explaining the causal structure of a BN applied to an environmental problem is relatively easy and more intuitive than most other modeling techniques we have applied. A bigger challenge is to explain the modeling of probability distributions and the role of the conditional probability tables (CPTs), which are often displayed only as arrows in diagrams. One area of active research in BNs is their application to stakeholder workshops and representing the concerns and understanding of stakeholders and experts for communicating technical information (Tiller et al. 2013; Carriger et al. 2018). In keeping with the spirit of the sessions, our intention is to provide information on the state of the art of BN practice in ERA, but also to welcome newcomers to these modeling tools and provide an equitable background for understanding the subsequent articles. A more detailed description can be found in, for example, Kaikkonen et al. (this issue).

## A BRIEF DESCRIPTION OF BAYESIAN NETWORKS

**Figure 2.**
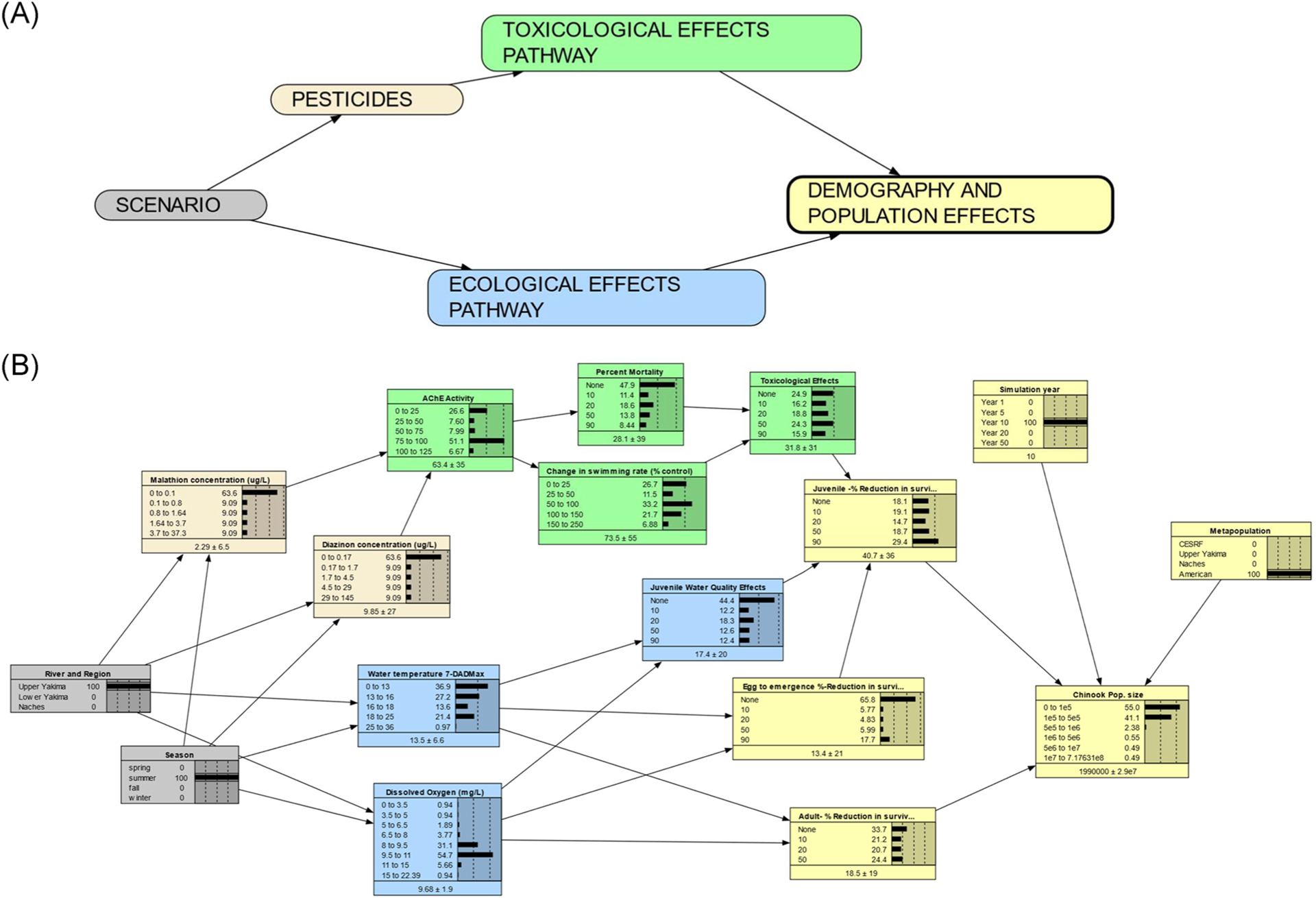
Example of a conceptual model **(A)** converted into a Bayesian network **(B)**, based on Mitchell et al. (this issue). The conceptual model describes the toxicological effect of pesticides in combination with ecological factors on populations of Chinook bass in South River, Virginia, USA. In technical terms, the conceptual model **(A)** is a directed acyclic graph consisting of nodes connected by arcs (arrows). The quantified model **(B)** can be viewed as a causal network of probability distributions. AChE = acetylcholinesterase; 7-DADMax = 7-d average of the daily maximum temperatures.

Invented in 1985 by Judea Pearl and colleagues (Pearl 1988), BNs represent a joint probability distribution among multiple variables in a graphical format. The graphical component of BNs consists of nodes that represent random variables and arcs (arrows) that connect the variables (Figure 1). The BN nodes and arrows form a directed acyclic graph, meaning that the network can have closed loops but no cycles (Figure 2). The nodes are usually defined by discrete states such as categories or intervals, although hybrid BNs can also contain continuous nodes (Moe et al. 2020). The arrows pointing into a node in a BN represent a causal relationship that is quantified in a CPT, which relates the probability for each state of the child node to each of the states of the parents (e.g., Carriger et al. 2016). The probability distribution of a child node is calculated from the probability distributions of its parent nodes combined with its CPT according to Bayes’ rule, which describes the probability of an event conditional on prior knowledge of conditions that might be related to the event (Kjærulff and Madsen 2008). Although BNs are used for causal modeling, the CPTs can be based on noncausal associations between variables (e.g., Carriger et al. this issue). The types of variables that can be represented are flexible but should include variables important to the questions addressed by the model, including confounding variables that would typically be controlled for in a randomized controlled trial, and that increase accuracy in prediction (Varis and Kuikka 1999).

Propagating uncertainties with a BN can be valuable for risk assessments that rely on multiple pieces of interacting evidence for characterizing risks from the fate and transport of stressors to predictions of potential adverse impacts. Sensitivity analysis is also robustly accommodated with BNs through information theory measures (Korb and Nicholson 2011), which helps to identify the influence of each variable on a target node in the BN. This can assist with identifying dominant variables dictating the risks to the assessment endpoints (Landis this issue; Piffady et al. this issue; Rachid et al. this issue).

**Figure 3.**
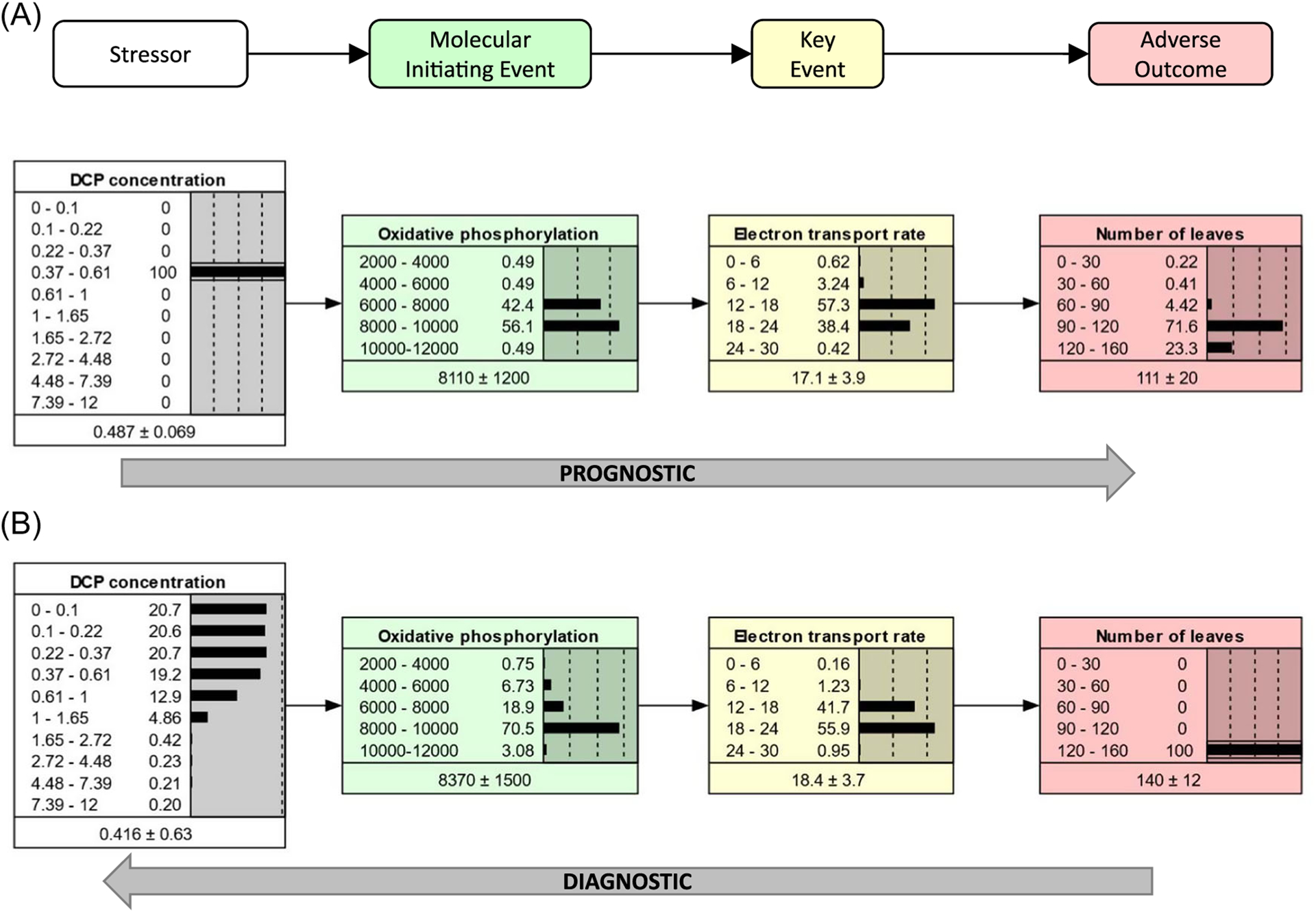
Examples of omnidirectional inference in a Bayesian network: Prognostic or predictive inference with observed stressor conditions and predicted effects **(A)**; diagnostic inference with stressor conditions predicted from observed effects **(B)**. The example represents an adverse outcome pathway in a plant exposed to the stressor 3,5-dichlorophenol (DCP), described by Moe et al. (this issue).

A unique property of BNs compared to, for example, dynamic process-based models, is that inference can be made in an omnidirectional fashion. The model can be run from cause to effect, that is, from the parent nodes in the direction of the arrow, for example, to predict biological effects of given chemical conditions (Figure 3A). However, a BN can also be run in the opposite direction from effect to cause, starting with one or more child nodes (Figure 3B). The BN can then back-calculate to the probability distributions of the cause (here, the stressor concentration) associated with the observed effects (here, the adverse outcome). Other examples of diagnostic inference can be found in Carriger and Newman (2012), Mitchell et al. (this issue), and Rachid et al. (this issue).

Using a BN makes the statistical and mechanistic assumptions in predictions and causal assessments transparent and tractable. The graphical engine of the BN makes it a transparent tool about what is being modeled and how it is being modeled. Several commercial and open-source software packages exist for using BNs, some of which are listed by Kaikkonen et al. (this issue). Each of these has unique features, but all can be useful for probabilistic causal modeling in assessment and management decision-making models.

## APPLICATIONS OF BAYESIAN NETWORKS TO ENVIRONMENTAL RISK ASSESSMENT

Bayesian networks have found their way into multiple everyday applications, including identifying oil locations, approving medical devices, medical diagnosis, operational risk management, legal profession, filtering emails for junk status, skill ranking for modern video games, and cell phone recognition (Fenton and Neil 2013; Pearl and Mackenzie 2018). Although relatively new, BNs have already been adopted in many fields of applied environmental science, including forestry (Marcot et al. 2001), fisheries (Uusitalo et al. 2012), and water resource management (Barton et al. 2008; Borsuk et al. 2012). The application of BNs to landscape-scale risk assessment was reviewed by Moe (2010). Landuyt et al. (2013) provided an overview for ecosystem service assessments and found their application to this area was still limited given the scope of ecosystem services, although their application in this area has been growing steadily since then (Stritih et al. 2020).

The qualitative structure of a BN can be used to capture the causal relationships in a conceptual model for ERA, whereas the quantitative part can capture uncertainties and nonlinear interactions between the conceptual model’s variables (Carriger and Parker 2021). The uncertainties regarding these relationships are captured in the CPTs (Ayre and Landis 2012; Moe et al. 2020). Thus, compared to the traditional use of conceptual models in ERA, BNs take the utility of the conceptual model one step further by allowing for quantitative information and incorporating the uncertainties in knowledge of causes and effects. When a conceptual model is placed into a BN format and the uncertainties among the relationships are quantified with conditional probabilities, the information can be used to predict risk as the probability of a given adverse effect (Figure 2A). Moreover, the parameterized BN can calculate the likely contribution of different causes to a given observed outcome (Figure 2B). Given the flexibility of their format, BNs can be adapted to many types of conceptual models used in ERA, representing both associations and causal relationships. For example, the traditional risk quotient (ratio of exposure vs effect) can be quantified as a full probability distribution instead of a single value (Carriger and Barron 2020). The causal key event relationships of qualitative adverse outcome pathway models can be quantified by CPTs (Moe et al. this issue). The cause–effect model recommended for ERA for gene drives (NASEM 2016) includes 5 interconnencted nodes (source, stressor, habitat, effects, and impacts), which can be expressed by BN modeling. The more complex multiple-stressor and multiendpoint systems described by the relative risk model framework (Landis this issue) can also be implemented in spatially structured BNs.

Causal and probabilistic modeling methods continue to gain in importance for capturing and representing the knowledge and data of important problems in risk assessment. For issues involving scenarios like climate change (Rachid et al. this issue), future chemical use (Piffady et al. this issue), and emerging contaminants (e.g., Landis this issue), the most critical questions are associated with uncertainty. For example, the predictions of future frequencies of severe storms, heat waves, and sea level rise require questions about the uncertainty of the frequency of occurrence and what proportion of the risk of an individual extreme event can be directly attributed to anthropogenic climate change (Pearl and Mackenzie 2018). Protecting communities and ecosystems will rely on understanding how uncertain our knowledge is on the rates of these future changes and how management interventions can mitigate future harms. The intuitive causal structure of BN models combined with the user-friendly graphical interface makes BNs a useful tool for evaluating these problems in a probabilistic manner, compared to other conventional probabilistic approaches to ERA such as joint probability curves (Verdonck et al. 2003).

## CONTENT OF THIS SPECIAL SERIES

This series provides examples of probabilistic ERA with BNs. The articles range from reviews to case study applications and explorations of theoretical aspects of BNs. Seven of the articles provide case studies that reflect the focus of the conference sessions on exploring where and how BNs are being used. Each of the case studies provides a unique network structure developed for multiple domains, including laboratory and field data, freshwater and marine risk problems, and endpoints that extend from single organisms (Moe et al. this issue) to populations (Mitchell et al. this issue), and human health and socioeconomic aspects (Cains and Henshel this issue; Rachid et al. this issue; Wade et al. this issue). Each of the case study applications also speak to broader ERA issues outside of their study domains. For example, the papers explore advanced methods such as machine learning (Carriger et al. this issue), temporally dynamic BNs (Rachid et al. this issue), influence diagrams involving decision and valuation nodes (Piffady et al. this issue), and adaptive management (Mitchell et al. this issue; Wade et al. this issue).

The series starts with a systematic literature review of BNs in ERA by Kaikkonen et al. (this issue). They examine the application of BN approaches to ERA in 72 papers published in the period 2004 to 2015, and analyze a range of aspects such as methods, participants, technical properties, environmental domain, stressors, and endpoints.

A retrospective of the implementation of BN methodology in the relative risk model framework (BN-RRM) is provided by Landis (this issue). Regional risk assessment with this framework has been adopted globally for a multitude of environmental problems. Landis provides a history of what drove advances in ERAs with BNs, where these advances have been applied and developed, and where they may go in the future. Many of the applications at the SETAC conference sessions were grounded in this framework for applying BNs to ERA, including 3 of the papers in this series (Cains and Henshel this issue; Mitchell et al. this issue; Wade et al. this issue).

Mitchell et al. (this issue) develop and apply a BN-RRM to assess the risk of pesticides in combination with other environmental stressors for Chinook salmon (*Oncorhynchus tshawytscha*) in the Yakima River Basin, Washington, USA. The multiple stressor impacts on the metapopulation dynamics of Chinook salmon populations are considered along with adaptive management recommendations based on diagnostic inference with multiple sources of evidence incorporated into the model. Wade et al. (this issue) apply the BN-RRM framework to a river system in South Africa. This article takes an ecosystem-based approach to ERA and examines the influence of stressors on water quality and quantity, habitat, and instream as well as riparian impacts on human and ecological uses. Cains and Henshel (this issue) present a holistic, systems engineering approach that incorporates consideration of both risk and resilience to examine the impacts of multiple stressors on socioecological systems. They demonstrate their approach in a coastal risk assessment for multiple threats, including contaminants, sea level rise, and storm surge. This example focuses on the risk to human communities and describes the parametrization and quantification for integrated risk and resilience assessments, paired with the BN-RRM as a proof of concept.

The next 2 papers in this series (Carriger et al. this issue; Moe et al. this issue) demonstrate that very different approaches can be used for parametrization of BNs (i.e., quantification of conditional probabilities). These 2 papers also exemplify that BN methodology can be applied to environmental problems at any spatial scale, ranging from molecular processes in plant cells to the global distribution of coral reefs. Moe et al. apply BNs for quantification of adverse outcome pathways (AOPs), which are structured representations of biological events leading to adverse effects. Adverse outcome pathways are considered highly relevant for risk assessment, from molecular to ecological outcomes, but in practice are limited by the lack of quantitative relationships (Conolly et al. 2017). Moe et al. (this issue) also exemplify how the uncertainties from laboratory data can be included in development of a quantitative AOP and provide an instructive use of Bayesian regression analysis for quantifying key event relationships and for developing CPTs more generally. Carriger et al. (this issue) use machine learning with BNs and spatial data to explore the relationships of indicators of coral reef biological status to local and global sources of stressors and management from marine protected areas. Although their model structures represent empirical associations learned from the data rather than causal relationships, a direct effects assessment was used to examine the implications of causal assumptions about some of the relationships in the models.

The 2 papers based on presentations for the EGU (Piffady et al. this issue; Rachid et al. this issue) provide examples of recent advancements in BN modeling with high relevance also for ERA. Piffady et al. (this issue) demonstrate the use of BNs in spatially explicit ERA for a country-wide assessment of risks from pesticides to surface waters based on basin-wide characteristics. The paper demonstrates the applicability of BNs to integrate interdisciplinary understanding, including soil science, hydrology, environmental chemistry, agronomy, GIS, and Bayesian modeling. The systems-based modeling approach offers an alternative to complex mechanistic models in situations where data are scarce and fast model run times are required for rapid decision support. The authors find that quantitative model validation may be difficult and hindered by data availability and present an alternative qualitative validation using stakeholder input. A user-friendly interface was built in R software (R Core Team 2018) to enable stakeholders to test management scenarios. The interface of BNs to spatial problems is an active area of research that was also explored in the previous special series (Johnson et al. 2012).

Rachid et al. (this issue) examine coastal problems from saltwater intrusion and provide a demonstration of a time-dynamic BN that explicitly considers the changes in risk and uncertainties over time. Dynamic BNs is an area of recent advancement in BN modeling (Marcot and Penman 2019). The integration of time in BNs remains an active research area, and to our knowledge Rachid et al. (this issue) present the most developed application of dynamic BNs in water quality modeling. One key feature of this case study is the adaptation of BNs to data-sparse but important problem areas, and the dynamic BN is especially insightful for examining sustainability of future water availability from the perspective of demand management and saltwater intrusion. This study finds that the importance of climate change for future water deficits is secondary to the impacts of demographic changes.

Sahlin et al. (this issue) conclude the series by providing an examination of uncertainty in BN modeling. They discuss how different sources of uncertainty—epistemic (knowledge based) and aleatory (stochastic)—can be handled within a BN framework, as well as the additional considerations in addressing and communicating uncertain knowledge.

## FUTURE APPLICATIONS OF BAYESIAN NETWORKS IN ENVIRONMENTAL RISK ASSESSMENT

The review paper by Kaikkonen et al. (this issue) provides recommendations for increasing future applications and value for decision making. Key knowledge gaps and directions for future research are identified, and several of these are addressed by articles in this special series. These include poor representation of cumulative risk and multiple stressors in traditional risk assessment models (Landis this issue), gaps in reporting of how stakeholders and experts were involved in model formulation (Piffady et al. this issue), how elicitation of subjective probabilities was undertaken (Piffady et al. this issue; Rachid et al. this issue), and what methods and criteria were used for discretization of continuous variables (Carriger et al. this issue; Rachid et al. this issue). The review found that networks using purely continuous data are very rare and hybrid networks using both continuous and discrete nodes are scarce, likely due to limited statistical distribution types in available BN modeling software. Model validation is often missing and is the subject of ongoing research (Carriger et al. this issue; Moe et al. this issue; Rachid et al. this issue). Use of decision and utility nodes in influence diagrams to inform adaptive management is underused, and transdisciplinary integration of ecological and socioeconomic aspects deserves further research (Cains and Henshel this issue; Rachid et al. this issue; Wade et al. this issue). Finally, combining BNs with spatial data is a growing area of research and development (Carriger et al. this issue; Piffady et al. this issue).

The applicability of BNs to environmental problems will still be challenged by various conceptual, methodological, and practical issues (Marcot 2017); such challenges also are addressed by most authors in this special series (e.g., Landis this issue; Rachid et al. this issue). Nevertheless, due to their capabilities to integrate probabilistic and causal modeling, increased and more advanced use of BNs in the future will benefit the practice of ERA. The capability of using a joint probability distribution to easily examine the potential impact of risk factors and stressors on the probability of negative outcomes for assessment endpoints allows them to naturally integrate into the ERA process and provide greater capabilities for analyzing the total or direct impacts of these variables on endpoints. Interpreting and communicating uncertainties is easier with BNs than with more traditional probabilistic methods used in ERA (Verdonck et al. 2003). Future scenarios can also be examined with the inferential capabilities of BNs, which makes them especially valuable for analyzing multiple stressor and climate change interactions with localized sources of stress. Bayesian networks are uniquely capable for addressing “what-if” questions, simulating hypothetical scenarios within a causal framework and quantifying the contribution of different variables to the observed outcome. Until recently, the latter has been largely the domain of randomized controlled trials, and BNs can address causal problems in situations where controlled experiments are not feasible or desirable (Moe et al. 2020). Furthermore, they allow causal inference in situations where data are sparse (Moe et al. this issue), or conversely, where observational data are more abundant but not collected as part of a rigorous experimental design (Carriger et al. this issue). Bayesian network modeling approaches have more advantages compared to other probabilistic approaches, including explicit representation of causal knowledge in the conceptual model (Moe et al. this issue), machine learning for identifying the best joint distribution to represent complex data (Carriger et al. this issue), and diagnostic inference for environmental conditions required to meet management goals (Mitchell et al. this issue; Rachid et al. this issue). Diagnostic inference is especially suited to retrospective risk assessments where past injuries are in question. The capabilities and limitations of these and other areas have not yet been fully explored in the context of ERA. We conclude that with the continued increase in use of BNs in ERA, best practices will become clearer and this tool will become even more useful for assessment and communication of environmental risk.

## Data Availability

There are no data directly associated with this paper. All data and results shown in the figures are available as Supplemental Data to the cited papers.
